# Manufacturing and Characterization of Dental Crowns Made of 5-mol% Yttria Stabilized Zirconia by Digital Light Processing

**DOI:** 10.3390/ma16041447

**Published:** 2023-02-09

**Authors:** Jae-Min Jung, Gyu-Nam Kim, Young-Hag Koh, Hyoun-Ee Kim

**Affiliations:** 1 Interdisciplinary Program in Precision Public Health, Korea University, Seoul 02841, Republic of Korea; 2 School of Biomedical Engineering, Korea University, Seoul 02841, Republic of Korea; 3 Department of Materials Science and Engineering, Seoul National University, Seoul 08826, Republic of Korea

**Keywords:** 3D printing, digital light processing, zirconia, dental crowns, strength

## Abstract

We herein report manufacturing of dental crowns made of 5-mol% yttria partially stabilized zirconia (5Y-PSZ) with desired mechanical properties, optical translucency and dimensional accuracy using digital light processing (DLP). To this end, all processing parameters were carefully controlled and optimized. First, 5Y-PSZ particles with a bimodal distribution were prepared via calcination of as-received granules and subsequent ball-milling and then used to formulate 5Y-PSZ suspensions with a high solid loading of 50 vol% required for high densification after sintering. Dispersant content was also optimized. To provide high dimensional accuracy, initial dimensions of dental crowns for 3D printing were precisely determined by considering increase and decrease in dimensions during photopolymerization and sintering, respectively. Photopolymerization time was also optimized for a given layer thickness of 50 μm to ensure good bonding between layers. A multi-step debinding schedule with a slow heating rate was employed to avoid formation of any defects. After sintering at 1500 °C for 2 h, 5Y-PSZ could be almost fully densified without noticeable defects within layers and at interfaces between layers. They had high relative densities (99.03 ± 0.39%) with a high cubic phase content (59.1%). These characteristics allowed for achievement of reasonably high mechanical properties (flexural strength = 625.4 ± 75.5 MPa and Weibull modulus = 7.9) and % transmittance (31.4 ± 0.7%). In addition, 5Y-PSZ dental crowns showed excellent dimensional accuracy (root mean square (RMS) for marginal discrepancy = 44.4 ± 10.8 μm and RMS for internal gap = 22.8 ± 1.6 μm) evaluated by the 3D scanning technique.

## 1. Introduction

Dental computer-aided design and computer-aided manufacturing (CAD/CAM) technology is still prevalent in the manufacturing of dental crowns made of yttria (Y_2_O_3_)-doped zirconia (ZrO_2_) ceramics [1,2]. However, this technique has several intrinsic limitations, including high consumption of cutting tools and great loss of discarded material during the subtractive machining process. Thus, the use of 3D printing of ceramics, which can construct 3D structures via direct addition of materials in a layer-by-layer fashion, has recently received a great deal of attention in modern dentistry to substitute for traditional dental CAD/CAM technology [3,4].

From the viewpoint of resolution and accuracy of 3D printing techniques, digital light processing (DLP) and stereolithography (SLA) have been most extensively investigated for the manufacturing of zirconia dental crowns [3,4,5,6,7,8,9,10,11,12,13,14,15]. These techniques can selectively photo-cure thin layers of zirconia suspensions, where zirconia particles are uniformly dispersed in photocurable monomers, using high-resolution UV engines according to pre-designed 2D images layer-by-layer [3,4]. 3D-printed zirconia crowns can then be heat-treated for debinding to remove organic phases and for sintering to densify zirconia particles. This approach can precisely replicate intricate 3D geometries of dental crowns designed for individual patients [3,4]. In the DLP process, a build platform can move downward to a predetermined position in a vat containing a zirconia suspension, and thus a uniform layer can be formed between the build platform and vat, which can be then photopolymerized by 2D images of UV light [7,8,12,13,15,16]. In addition, recoating systems can be integrated into DLP machines (e.g., lithography-based ceramic manufacturing (LCM)), in order to mechanically spread ceramic suspensions uniformly just above the bottom of a vat [5,14]. This approach is very useful for ceramic suspensions with high viscosities and low flowablility. However, this bottom-up approach has a potential risk of interfacial failure between photopolymerized layers, caused during detachment of newly photopolymerized layers from a polymer film attached to the bottom of a vat, and frequent damage of the film. On the other hand, in the SLA process, a recoater can directly spread a ceramic suspension onto a previously photopolymerized layer, thus eliminating issues related to the bottom-up approach [9,10,11,14]. However, this top-down approach requires a large number of ceramic suspensions to fill a vat and the leveling process to ensure uniform thickness is troublesome. To avoid these limitations, thin layers formed onto polymer films can be supplied continuously, which employ a basic concept of ceramic tape-casting [17]. In addition, our group recently developed a new type of top-down DLP process by employing specifically designed feeding and recoating systems, where ceramic suspensions can be extruded through a nozzle at a controlled rate and then uniformly spread onto previously photopolymerized layers by a recoater [6]. A number of recent works have demonstrated that DLP and SLA techniques could offer outstanding dimensional accuracies comparable to CAD/CAM [10,11,12,13,14,15,18]. However, 3D-printed zirconia objects often suffer from imperfections, including pores within layers and weak interfaces between layers, causing severe reductions in mechanical properties [5,6,18,19,20,21,22,23,24,25] and optical translucency [6,22].

The generation of these imperfections is mainly due to a difficulty in formulation of zirconia suspensions with high solid loadings required for almost full densification. More specifically, as the solid loading increases, the viscosities of zirconia suspensions increase dramatically, thus making it troublesome to uniformly disperse a large amount of zirconia particles in photocurable monomers [26]. Thus, pores and voids will be formed in sintered zirconia ceramics due to incomplete densification of zirconia particles when solid loadings are not sufficiently high. In addition, weak interfaces between layers will be caused by sedimentation of zirconia particles in photocurable monomers during the 3D printing process. Thus, to newly formulate zirconia suspensions with the desired properties, several factors, including particle size and its distribution [26,27], type and number of dispersants [28] and compositions of photocurable vehicles containing reactive [9,29,30,31] and non-reactive diluents [6,29,32,33,34], should be carefully optimized to provide reasonably low viscosities for uniform dispersion of zirconia particles at high solid loadings. In addition, the mechanical and optical performances of zirconia dental crowns should also be also strongly affected by compositions of zirconia ceramics used to prepare zirconia suspensions. In this respect, 5 mol% yttria partially stabilized zirconia (5Y-PSZ) is very promising since it can offer much higher optical translucency than other zirconia ceramics with lower yttria contents or −3 mol% yttria stabilized tetragonal zirconia polycrystal (3Y-TZP) and 4 mol% yttria partially stabilized zirconia (4Y-PSZ) [35,36,37], while still providing reasonable mechanical properties for dental crown applications. However, the manufacturing of 5Y-PSZ dental crowns using DLP and SLA has been rarely reported.

Thus, we herein carefully optimized processing parameters for DLP process to manufacture 5Y-PSZ dental crowns with the desired mechanical properties, optical translucency and dimensional accuracies. In particular, special care was paid to formulate 5Y-PSZ suspensions with high solid loading (50 vol%) and uniform distribution of particles in photocurable vehicles. To this end, as-received 5Y-PSZ granules were calcined at 1000 °C for 1 h and then crushed into submicron-sized 5Y-PSZ particles via ball-milling. Dispersant content was also optimized to obtain the highest solid loading with reasonably low viscosities for the DLP process. Photocurable 1,6-hexanediol diacrylate (HDDA) monomer was blended with decalin used as the diluent [6]. A custom-built DLP machine was employed to manufacture 5Y-PSZ specimens and dental crowns since it could directly deposit thin layers of 5Y-PSZ suspensions onto previously photocured layers by a recoater, thus eliminating imperfections at interfaces between layers often observed in conventional DLP [6]. To achieve excellent dimensional accuracy, several processing parameters, including initial designs of dental crowns, layer thickness of 5Y-PSZ suspensions for sequential photocuring process, and UV illumination time were precisely controlled. A schedule for the debinding process to remove organic phases (photocured HDDA, dispersant, and photo-initiator) was carefully established by considering the thermal decomposition behavior of as-built 5Y-PSZ specimens. Subsequently, 5Y-PSZ specimens and dental crowns were sintered at 1500 °C for 2 h. Densification behaviors (e.g., shrinkage, relative density and microstructure) and crystalline phases of 5Y-PSZ were characterized using several analyzing tools. Mechanical properties were characterized by three-point flexural strength tests. Optical transmittances were evaluated by spectrophotometry. Dimensional accuracies of 5Y-PSZ dental crowns were examined in terms of marginal and internal discrepancies using 3D scanning.

## 2. Materials and Methods

### 2.1. Preparation of Submicron-Sized 5Y-PSZ Particles

**Figure 1:**
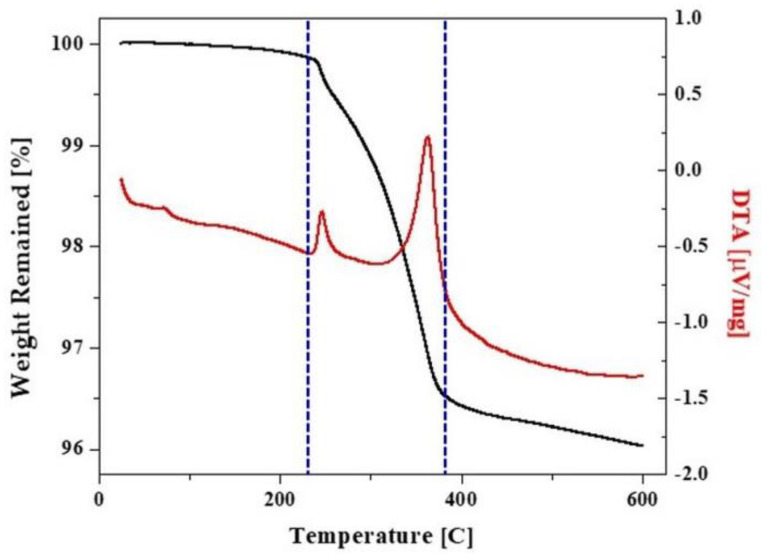
TG-DTA curve observed for as-received 5Y-PSZ granules as a function of temperature. The black and red lines represent TG and DTA results, respectively.

5 mol% yttria-partially stabilized zirconia (5Y-PSZ, Zpex Smile) granules were purchased from Tosoh Co. (Tokyo, Japan). To determine the calcination temperature for removing polymeric binder within granules and inducing partial necking between nanoparticles, thermal decomposition behavior of as-received granules was examined by thermogravimetry (TG)-differential thermal analysis (DTA) (STA 8122, Rigaku Corp., Tokyo, Japan). For this evaluation, a batch of granules was heated up to 600 °C at a heating rate of 5 °C/min in air and its weight loss was recorded as a function of temperature, as shown in Figure 1. Considerable weight loss was observed at the temperature range of ~240–390 °C, indicating extensive thermal decomposition of polymeric binder within granules. Above 400 °C, weight loss was very low. Thus, to prepare 5Y-PSZ suspensions with high solid loadings, as-received granules were calcined at 1000 °C for 1 h to completely remove polymeric binder within granules and induce partial necking between nanoparticles. In addition, for comparison purpose, as-received granules were calcined at a low temperature of 500 °C for 1 h to preserve the original structure of nanoparticles without necking.

Calcined granules were then vigorously crushed into smaller particles by ball-milling at a rotation speed of 90 rpm for 24 h using zirconia balls as media. Morphologies and size distributions of as-received granules, calcined granules and crushed particles were characterized using a field emission scanning electron microscope (FE-SEM; JSM-6701F, JEOL Techniques, Tokyo, Japan) and a laser particle size analyzer (CILAS 1090; Orleans, France), respectively.

### 2.2. Constituents of Photocurable 5Y-PSZ Suspensions

Table 1 summarizes the constituents of photocurable 5Y-PSZ suspensions with a solid loading of 50 vol% used for manufacturing dental crowns by our DLP process. Unless otherwise notified, all reagents were used as purchased. To obtain reasonably low viscosities at high solid loadings, 1,6-hexanediol diacrylate (HDDA; Sartomer, Exton, PA, USA) and decalin (Sigma Aldrich, St. Louis, MO, USA) were employed as the photopolymerizable monomer and diluent, respectively [26]. An alkylol-ammonium salt of a copolymer with acidic groups (DISPERBYK-180; BYK-Chemie GmbH, Wesel, Germany) was used as a dispersant. Diphenyl(2,4,6-trimethylbenzoyl) phosphine oxide (TPO; Sigma Aldrich, St. Louis, MO, USA) was used as a photo-initiator.

### 2.3. Preparation of 5Y-PSZ Suspensions

For preparing 5Y-PSZ suspensions, a solution of 65 wt% HDDA-35 wt% decalin was used as a photocurable liquid medium. Predetermined amounts of HDDA, decalin, dispersant (2 wt% with respect to the 5Y-PSZ particle content) and zirconia balls as a media for vigorous mixing were added into plastic bottles. The mixtures were then blended by a planetary centrifugal mixer (Hantech Co. Ltd., Ansan-si, Gyeonggi-do, Republic of Korea) for 10 min at a rotation speed of 1000 rpm. Subsequently, submicron-sized 5Y-PSZ particles were added into liquid medium and then vigorously mixed by the planetary centrifugal mixer for 30 min. This mixing process was repeated three times using predetermined amounts (1/2, 1/4 and 1/4 of the total weight in sequence) of particles, in order to achieve uniform dispersion of particles in liquid mediums. Three different types of 5Y-PSZ suspensions with a range of solid loadings (44 vol%, 48 vol% and 50 vol%) were prepared to examine their rheological behaviors. For photocuring by DLP, 2 wt% of photo-initiator with respect to the HDDA content was mixed with 5Y-PSZ suspensions for 10 min.

### 2.4. Rheological Behavior Evaluations of 5Y-PSZ Suspensions

Rheological behaviors of 5Y-PSZ suspensions were evaluated by measuring their apparent viscosities as a function of shear rate (0.1 s^−1^–200 s^−1^) using a cone/plate viscometer (Brookfield DV3T, Brookfield Engineering Laboratories, Inc., Middleborough, MA, USA). To optimize the dispersant content, 5Y-PSZ suspensions (solid loading = 44 vol%) prepared using a range of dispersant contents (1 wt%, 2 wt%, 3 wt% and 4 wt%) were examined. To examine the effect of solid loading on rheological behavior, 5Y-PSZ suspensions with different solid loadings (44 vol%, 48 vol% and 50 vol%) were examined.

### 2.5. Custom-Built DLP Process

**Figure 2:**
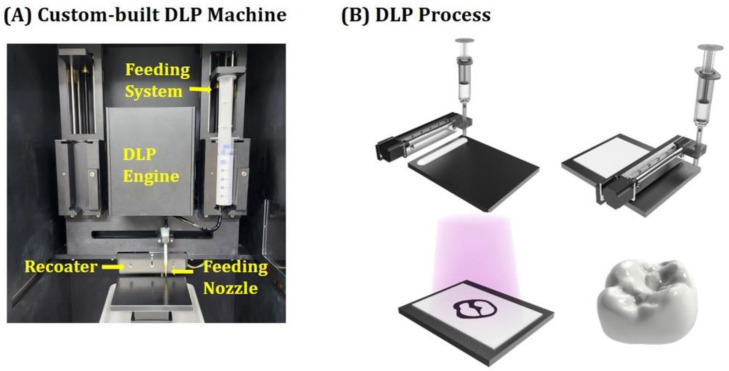
(**A**) Optical image of our custom-built DLP machine and (**B**) schematics showing the DLP process used for manufacturing 5Y-PSZ dental crowns.

5Y-PSZ specimens and crowns were manufactured using our custom-built DLP machine employing a specially designed feeding and recoating systems (Veltz3D, Incheon, Republic of Korea), as shown in Figure 2A, whose basic operation principle was proposed in our previous report [6]. To offer high accuracy for dental crown applications, a digital micromirror device (DMD) light engine with 1920 × 1080 pixels was employed, which could illuminate a UV power of ~14.95 mW/cm^2^ at a peak wavelength of ~405 nm. Schematics showing the DLP process used for manufacturing 5Y-PSZ dental crowns are illustrated in Figure 2B. Our DLP machine could extrude highly concentrated 5Y-PSZ suspensions in a controlled manner using a stepper motor and then spread them uniformly using a recoater. In addition, after selective photopolymerization of the first layer, the second fresh layer could be directly deposited onto the first layer. This approach could eliminate a potential risk of interfacial delamination between layers that can occur during detachment of photopolymerized layers from a vat in the conventional DLP process.

To ensure complete photocuring of 50-micron-thick layers of 5Y-PSZ suspensions and good bonding between layers, fresh layers were photocured for various time periods (1 s–10 s) and thicknesses of photocured layers were measured by a micrometer. In addition, to obtain high dimensional accuracy for dental crown applications, increases in dimensions via UV scattering by ceramic particles during the photocuring process were evaluated quantitatively [38,39,40]. To this end, disk-shaped regions with different diameters (100 μm–1000 μm) in 50-μm-thick-layers of 5Y-PSZ suspensions were photopolymerized for 3 sec, after which photocured disks were washed with ethanol and then their diameters were calculated by FE-SEM. The degree of discrepancy between designed and printed diameters was computed, representing broadening.

### 2.6. Manufacturing of 5Y-PSZ Specimens and Dental Crowns

5Y-PSZ specimens and dental crowns were manufactured by our DLP process. To this end, 50-micron-thick layers of 5Y-PSZ suspensions with a solid loading of 50 vol% were photocured for 3 s. Two types of specimens (rectangular bars and disks) were manufactured to estimate mechanical properties and optical translucency of 5Y-PSZ dental crowns. To achieve high accuracy, initial dimensions of dental crowns (c.f., Figure 2B) were precisely determined by considering opposing changes in dimensions during the entire process—increase due to UV scattering by 5Y-PSZ particles during photopolymerization process and decrease due to densification of 5Y-PSZ particles via sintering at high temperatures. Based on our preliminary studies, we employed an enlargement of ~25.2% and 24.5% in the x-y and z-directions, respectively, in order to design an initial CAD model for the DLP process.

**Figure 3:**
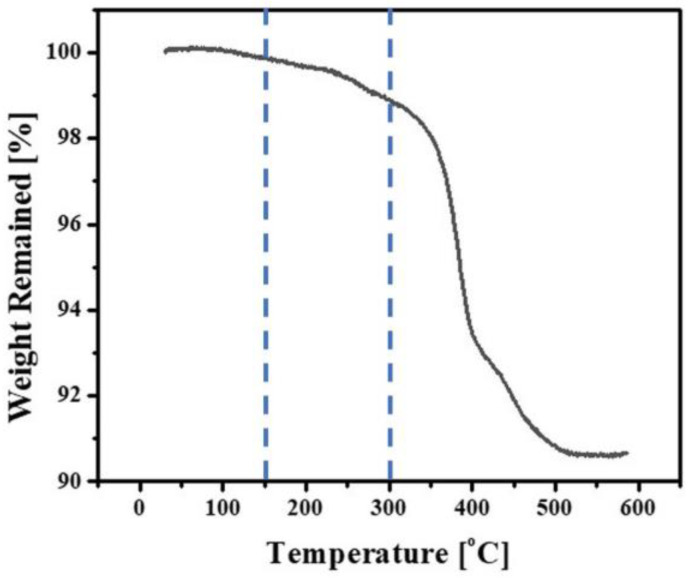
TGA curve observed for an as-printed 5Y-PSZ sample as a function of temperature. The dotted lines at 150 °C and 300 °C represent starting temperatures for thermal decomposition of organic phases and photopolymerized HDDA, respectively.

As-printed 5Y-PSZ specimens and dental crowns were removed from build platforms and then rinsed with ethanol several times to remove uncured suspensions, after which green specimens and dental crowns underwent heat-treatment for debinding and sintering. To design a schedule for debinding, the thermal decomposition behavior of a green specimen was characterized by thermogravimetric analysis (TGA; STA 449 F3 Jupiter; Netzsch GmbH, Selb, Germany). For this evaluation, a specimen was heated up to 600 °C at a heating rate of 10 °C/min in air and its weight loss was recorded as a function of temperature. A representative TGA curve is shown in Figure 3. Thermal decomposition started at a temperature of 150 °C due to removal of diluent, dispersant, and photo-initiator. Above 300 °C, remarkable weight losses were observed due to intensive removal of photopolymerized HDDA.

On the basis of the TGA analysis, a multi-step debinding schedule was carefully designed, where slow heating rates below 470 °C were employed to remove photopolymerized HDDA and organic phases without formation of defects such as cracks within layers and at interfaces between layers (Table 2). After debinding, 5Y-PSZ specimens and dental crowns were densified via sintering at 1500 °C for 2 h.

### 2.7. Characterization of as-Printed and Sintered 5Y-PSZ Specimens

3D geometries of as-printed and sintered 5Y-PSZ specimens and dental crowns were examined by optical microcopy. Microstructures of as-printed and sintered 5Y-PSZ specimens and dental crowns were characterized by FE-SEM. External and internal structures of dental crowns were examined by micro-computed tomography (μ-CT; SkyScan1273, Bruker Corp., Billerica, MA, USA). A voxel size of 40 μm was used for image analyses.

Shrinkages after sintering at 1500 °C for 2 h were computed by comparing dimensions of sintered specimens to those of as-manufactured specimens. Densities of sintered specimens were measured using Archimedes’ principle. Their relative densities were computed based on measured densities and theoretical density of 5Y-PSZ (6.034 g/cm^3^) [41]. However, it should be noted that theoretical density of 5Y-PSZ should be affected by the contents of tetragonal and cubic phases. Grain size was measured by the linear intercept method. In addition, an image analysis program (MIPAR, MIPAR Image Analysis, Columbus, OH, USA) was used to roughly examine distribution of grain sizes.

Crystalline phases of sintered 5Y-PSZ specimens were characterized using an X-ray diffractometer (DMAX-2500, Rigaku Corporation, Tokyo, Japan) using Cu Kα radiation. Observed peaks were then identified by considering diffraction patterns (diffraction angles and relative intensities) of tetragonal (JCPDS card no. 01-079-1767) and cubic (JCPDS card no. 01-081-1550) phases of zirconia. Rietveld refinement using X’Pert Highscore Plus software 3.0 (Malvern Panalytical, Worcestershire, UK) was used to calculate contents of tetragonal and cubic phases coexisting in 5Y-PSZ.

In addition, an energy dispersive X-ray spectroscope (EDS; Horiba, Ltd., Kyoto, Japan) attached to a FE-SEM (SU-70, Hitachi Ltd., Hitachi, Japan) was used to examine chemical compositions of grains with tetragonal and cubic phases. To this end, EDS spot analyses with a spot size of 0.4 μm were conducted in two different types of grains (large and small grains) and 14 points were tested for each grain.

### 2.8. Mechanical Properties Evaluation

To estimate mechanical functions of 5Y-PSZ dental crowns, fracture strength and Weibull modulus of sintered specimens were measured using three-point flexural strength tests according to ISO 6872 (Dentistry—Ceramic materials) [42,43,44]. Rectangular specimens were grounded to obtain dimensions of 3 mm × 4 mm × 25 mm and then polished to ensure smooth surfaces. A constant crosshead speed of 1 mm/min was applied to the center of each specimen using a screw-driven load frame (Instron 4467, Instron, MA, USA). Load versus displacement curves were recorded until specimens were fractured. Flexural strengths were then computed by considering loads at fracture and dimensions (thicknesses and widths) of specimens. Twelve specimens were tested to obtain mean and standard deviation. In addition, a Weibull plot of failure probability (*P_f_*) versus measured flexural strengths (σ) was constructed to determine Weibull modulus (*m*) representing mechanical reliability of 5Y-PSZ specimens and characteristic strength (*σ_o_*) [45].

### 2.9. Optical Translucency Evaluation

To estimate optical translucency of 5Y-PSZ dental crowns, a computer-controlled spectrophotometer (CM-3600A, Konica Minolta, Inc., Osaka, Japan) was used to directly measure optical transmittances of sintered disks. For these evaluations, the sintered disks were slightly ground to obtain a thickness of 1 mm and then polished to ensure flat, smooth surfaces. Spectral transmittance data over the spectral range from 360 nm to 740 nm at 10 nm intervals were collected. % transmittance was computed according to the standard (CIE standard illuminant D65) [46]. Three samples were tested to obtain mean and standard deviation.

### 2.10. Dimensional Accuracy Evaluation

To examine potentials of 3D-printed 5Y-PSZ dental crowns for clinical use, their dimensional accuracies in terms of marginal and internal fits were closely examined by a 3D scanning technique [13,47,48]. For these evaluations, 5Y-PSZ dental crowns were scanned by a dental scanner (Medit T500, Medit Corp., Seoul, Republic of Korea) having a precision of 7 μm. Scanned data were recorded using a scan software (colLab 2017 v2.0.0.4, Medit Corp., Seoul, Republic of Korea). Color maps were then generated by superimposing scanned data of 5Y-PSZ dental crowns over original CAD data and then analyzed using a 3D analysis software program (Geomagic ControlX 2020.1 ver.; 3D Systems, Inc., Rock Hill, SC, USA). Blue and red colors indicate negative and positive deviations compared to the reference (original CAD data), respectively, while green color indicate deviations within ±50 μm. Internal discrepancies between original CAD design and manufactured 5Y-PSZ dental crowns were quantified in terms of root mean square (RMS) obtained from the entire inner area of dental crowns [49,50]. Five specimens were tested to obtain mean and standard deviation.

### 2.11. Statistical Analysis

Statistical analysis was carried out using one-way analysis of variance (ANOVA) with MATLAB (The MathWorks, Inc., Natick, MA, USA) and Tukey–Kramer Post Hoc Test (OrginLab Corp., Northampton, MA, USA). *p* values less than 0.05 were considered statistically significant.

## 3. Results and Discussion

### 3.1. Morphologies and Size Distributions of 5Y-PSZ Granules and Particles

**Figure 4:**
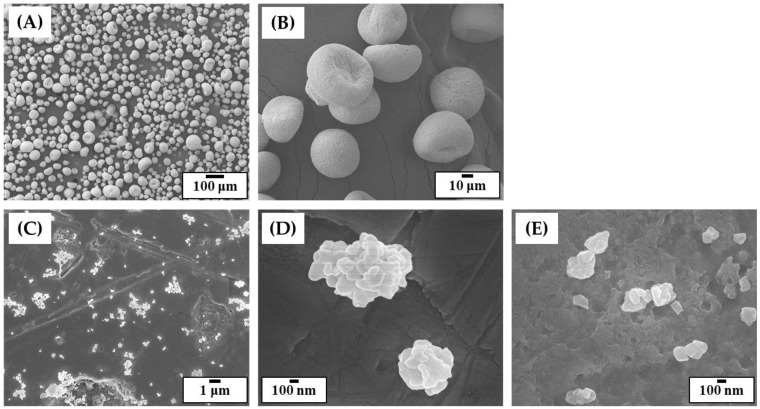
Representative FE-SEM images showing as-received 5Y-PSZ granules (**A**), granules calcined at 1000 °C (**B**), crushed particles obtained after ball-milling of granules calcined at 1000 °C (**C**,**D**), and crushed particles obtained after ball-milling of granules calcined at 500 °C (**E**).

To manufacture dental crowns, commercially available 5Y-PSZ granule, which has been widely used to produce zirconia blanks for dental CAD/CAM, was employed owing to its high translucency [36]. However, as-received granules showed very large sizes with several tens of microns (Figure 4A). In addition, they could not be crushed into smaller particles because of a small amount (~4 wt%) of polymeric binder. Thus, as-received granules were heat-treated to 1000 °C for 1 h to completely remove polymeric binder, while their spherical morphologies and sizes changed negligibly (Figure 4B). After ball-milling of calcined granules, fine particles were obtained (Figure 4C). In addition, 5Y-PSZ nanoparticles were partially bonded together due to calcination at a relatively high temperature of 1000 °C (Figure 4D). On the other hand, when granules calcined at a lower temperature of 500 °C were ball-milled, they were crushed into very fine particles (Figure 4E) since nanoparticles were weakly bonded together. This finding suggests that a high calcination temperature of 1000 °C could effectively reduce the specific surface area of particles, thus being beneficial to achieve high solid loadings with reasonably low viscosities [26]. However, it should be noted that particle size distribution can also strongly affect viscosities of 5Y-PSZ suspensions [25,51,52,53].

**Figure 5:**
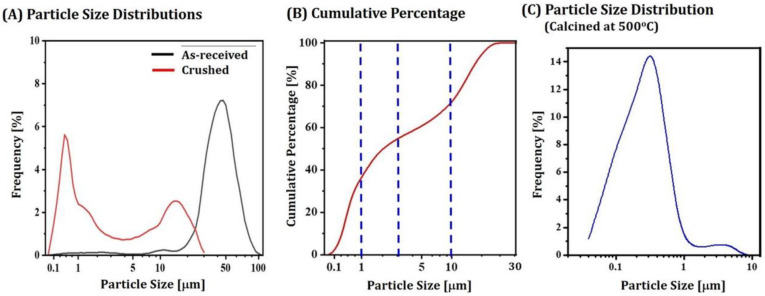
(**A**) Size distributions of as-received 5Y-PSZ granules and crushed 5Y-PSZ particles obtained after ball-milling of granules calcined at 1000 °C (**B**) cumulative particle size distribution of percentage of crushed 5Y-PSZ particles obtained after ball-milling of granules calcined at 1000 °C, and (**C**) size distributions of crushed particles obtained after ball-milling of granules calcined at 500 °C. The dotted lines in Figure 5B represent particle sizes of 1 μm, 3 μm, and 10 μm.

Size distributions of as-received 5Y-PSZ granules and crushed particles obtained via ball-milling of granules calcined at 1000 °C were characterized by a laser particle size analyzer. Results are shown in Figure 5A. As-received granules showed relatively large sizes ranging from 5 μm to 100 μm. The mean size was as large as ~50 μm. On the other hand, crushed particles obtained after calcination at 1000 °C and subsequent ball-milling showed much smaller sizes. In addition, they showed a bimodal distribution with two peaks at ~0.3 μm and ~15 μm. A cumulative particle size distribution of crushed particles is displayed in Figure 5B. The fractions of particles with size ranges of 0.1–1 μm, 1 μm–3 μm, 3 μm–10 μm and 10 μm–30 μm were 34.04%, 19.08%, 16.71%, and 29.42%, respectively. This finding suggests that calcined granules could be efficiently crushed into smaller particles by ball-milling. In addition, a wide particle size distribution could be beneficial for preparing 5Y-PSZ suspensions with reasonable viscosities at high solid loadings since voids formed between large particles could be filled by smaller particles [25,52]. On the other hand, particles obtained via ball-milling of granules calcined at a relatively low temperature of 500 °C showed much smaller sizes (<1 μm) with a unimodal distribution (Figure 5C).

### 3.2. Effect of Particle Sizes on Rheological Behaviors of 5Y-PSZ Suspensions

**Figure 6:**
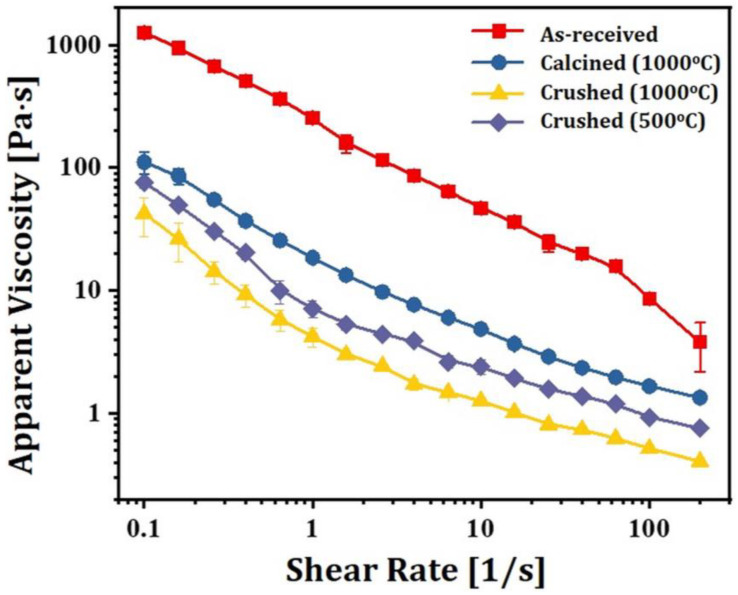
Apparent viscosities as a function of shear rate observed for 5Y-PSZ suspensions prepared using as-received, granule calcined at 1000 °C, and crushed particles obtained via ball-milling of granules calcined at 1000 °C and 500 °C (solid loading = 44 vol%).

To examine the effect of particle size distribution on rheological behaviors of 5Y-PSZ suspensions, four different types of suspensions were prepared using as-received granules, granules calcined at 1000 °C and crushed particles obtained via ball-milling of granules calcined at 1000 °C and 500 °C, and 2 wt% of dispersant was employed for uniform dispersion of particles in photocurable vehicles. Apparent viscosities of 5Y-PSZ suspensions were measured as a function of shear rate using a cone/plate viscometer. Results are displayed in Figure 6. All suspensions showed shear thinning behaviors, where viscosity decreased with an increase in shear rate. However, when as-received granules were used, suspensions showed very high viscosities due to large size of granules. On the other hand, granules calcined at 1000 °C could be partially crushed during vigorous mixing for preparation of suspensions, thus resulting in lower viscosities than as-revived granules. In addition, when crushed particles obtained via ball-milling of granules calcined at 1000 °C were used, significantly lower viscosities were observed. This reduction in viscosity was mainly attributed to a broad particle size distribution (c.f., Figure 5B), and thus small particles could fill voids formed between large particles [26,52]. However, when a low calcination temperature of 500 °C was used, nanoparticles within granules were weakly bonded together, and thus very fine particles could be obtained by ball-milling (c.f., Figure 5C). Thus, 5Y-PSZ suspensions prepared using these fine particles showed higher viscosities than those prepared using particles obtained via ball-milling of granules calcined at a higher temperature of 1000 °C. This finding suggests that it is crucial to prepare 5Y-PSZ particles with proper size ranges by optimizing the calcination temperature (c.f., 1000 °C in this study) and ball-milling process, which allows for achievement of high solid loadings with reasonably low viscosities.

### 3.3. Optimization of Dispersant Content

**Figure 7:**
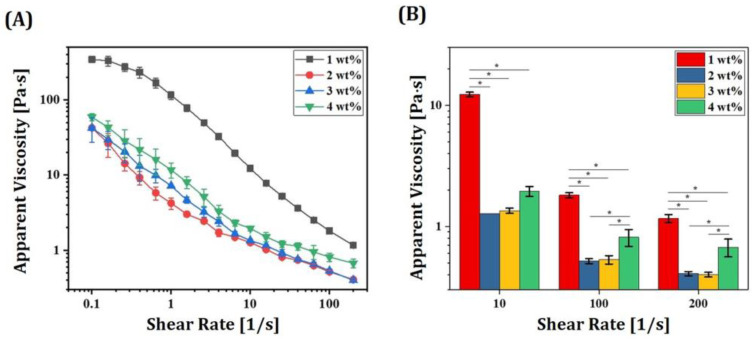
(**A**) Apparent viscosities as a function of shear rate observed for 5Y-PSZ suspensions with a solid loading of 44 vol% prepared using a range of dispersant contents (1 wt%, 2 wt%, 3 wt%, and 4 wt%) and (**B**) apparent viscosities measured at various shear rates (* *p* < 0.05).

To obtain high solid loadings with low viscosities, a solution of low-viscosity HDDA as a photopolymerizable monomer and decalin as a diluent was employed as a liquid medium. In addition, to induce strong repulsion between 5Y-PSZ particles, a kind of an alkylol-ammonium salt of a copolymer with acidic groups was employed as a dispersant [6]. To optimize dispersant content in relation to particle content, 5Y-PSZ suspensions were prepared using a range of dispersant contents (1 wt%, 2 wt%, 3 wt% and 4 wt%). For these tests, a solid loading of 44 vol% was used. Figure 7A shows apparent viscosities as a function of shear rate observed for different dispersant contents. All 5Y-PSZ suspensions showed a decrease in viscosity with an increase in shear rate. Such shear thinning behaviors are highly beneficial for the DLP process [51]. When the dispersant content increased from 1 wt% to 2 wt%, remarkable reductions in viscosity were observed over all shear rates (Figure 7B). However, with further increases in dispersant content (3 wt% and 4 wt%), viscosity increased. This finding suggests that 2 wt% of the dispersant used in this study could sufficiently adsorb on entire surfaces of 5Y-PSZ particles and induce sufficient repulsion between particles, thus offering significantly reduced viscosities. However, when excessive dispersant was used, viscosity increased due to an increase in thickness of the adsorbed layer [26].

### 3.4. Optimization of Maximum Solid Loading

**Figure 8:**
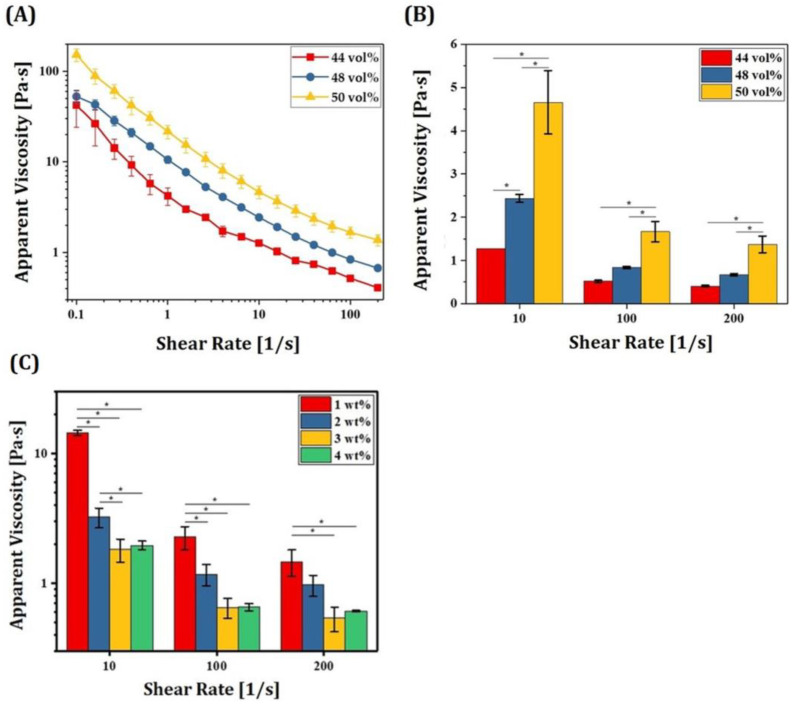
(**A**) Apparent viscosities as a function of shear rate observed for 5Y-PSZ suspensions prepared using a range of solid loadings (44 vol%, 48 vol% and 50 vol%), (**B**) apparent viscosities at various shear rates observed for 5Y-PSZ suspensions (* *p* < 0.05), and (**C**) apparent viscosities of 5Y-PSZ suspensions with a solid loading of 50 vol% prepared using a range of dispersant contents (* *p* < 0.05).

For manufacturing zirconia dental crowns with desired mechanical properties and optical translucency, it is crucial to achieve high densification with negligible defects after sintering at high temperatures. To this end, 5Y-PSZ suspensions should have high solid loadings since highly concentrated ceramic particles can be densified more favorably, while reasonably low viscosities should be maintained to allow for the formation of thin, uniform layers by a recoater for sequential DLP processes. 5Y-PSZ suspensions were prepared using a range of solid loading (44 vol%, 48 vol% and 50 vol%) and their rheological behaviors were characterized to determine the maximum solid loading obtainable using our compositions (c.f., Table 1). The same dispersant content of 2 wt% was used for all suspensions. Apparent viscosities as a function of shear rate observed for 5Y-PSZ suspensions prepared using a range of solid loadings are shown in Figure 8A. Shear thinning behaviors with a decrease in viscosity with an increase in shear rate were observed for all 5Y-PSZ suspensions. On the other hand, higher solid loading resulted in higher viscosities over all shear rates (Figure 8B), which is a common phenomenon of ceramic suspensions for colloidal processes [26,27,53]. However, 5Y-PSZ suspensions with the highest solid loading of 50 vol% showed reasonably low viscosities (1.37 ± 0.19 Pa·s) at a high shear rate of 200 s^−1^, suggesting that they could be spread uniformly by a recoater for sequential DLP processes. In addition, reasonably high viscosities (4.66 ± 0.73 Pa·s) were observed at a low shear rate of 10 s^−1^ and thus fresh layers of 5Y-PSZ suspensions deposited on previously photopolymerized layers could be maintained fairly during photopolymerization. Effect of dispersant content on apparent viscosities of 5Y-PSZ suspensions with a solid loading of 50 vol% was also examined, as shown in Figure 8C. At a low shear rate of 10 s^−1^, 3 wt% showed lower viscosities than 2 wt%. However, at high shear rates (100 s^−1^and 200 s^−1^), 2 wt% and 3 wt% showed no significant differences in viscosity. It should be noted that HDDA content decreases with an increase in dispersant content, thus resulting in lower green strength after photopolymerization. Thus, it is reasonable to suppose that 2 wt% of dispersant used in this study is sufficient to uniformly disperse 5Y-PSZ particles and offer reasonably high green strengths. However, when a higher solid loading of 52 vol% was used, 5Y-PSZ suspensions showed very high viscosities (data now shown here). In addition, air was often trapped within layers during DLP. Thus, a solid loading of 50 vol% was employed for manufacturing 5Y-PSZ specimens and dental crowns, in order to achieve almost full densification without imperfections after sintering at 1500 °C for 2 h.

### 3.5. Optimization of Photocuring Process for DLP

**Figure 9:**
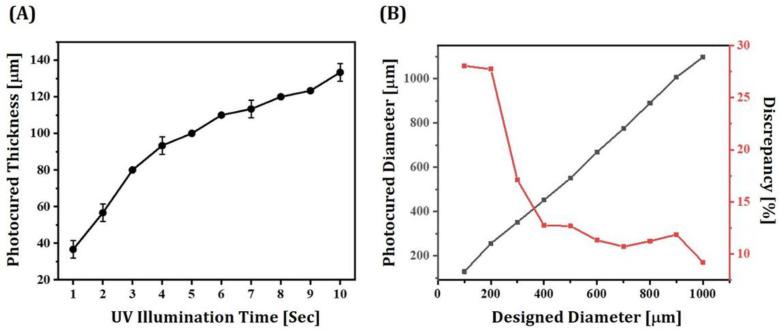
(**A**) Thicknesses of photocured layers obtained using a range of UV illumination time (1 s–10 s) and (**B**) diameters of photocured disks in regard to designed diameters and percent (%) discrepancies between measured and initial values. The black and red symbols in Figure 9B represent photocured diameters and discrepancies, respectively.

To completely consolidate layers of 5Y-PSZ suspensions with high dimensional accuracy, UV illumination time for photocuring should be carefully determined. Thus, layers of a 5Y-PSZ suspension with a solid loading of 50 vol% were photocured for a range of illumination time periods (1 s to 10 s). Photocured layers were then washed with ethanol and their thicknesses were measured using a micrometer. Figure 9A shows measured thicknesses of photocured layers as a function of UV illumination time. The photocured thickness increased almost linearly from 37.8 ± 1.2 μm to 93.2 ± 1.2 μm with an increase in UV illumination time from 1 s to 4 s. A UV illumination time of 2 s showed photocured thickness (56.7 ± 4.7 μm) slightly thicker than layer thickness (50 μm) of 5Y-PSZ suspensions, which would have a risk of imperfections between photocured layers particularly for manufacturing of complicated structures (e.g., dental crowns). Thus, to ensure strong bonding between photocured layers, a UV illumination time of 3 sec, which could result in a photocured thickness of 81.4 ± 1.0 μm, was employed for the DLP process.

For manufacturing 5Y-PSZ dental crowns using the DLP technique, special care should be paid to determine initial dimensions of dental crowns since there should be enlargement and shrinkage during photopolymerization and sintering, respectively. More specifically, when predetermined regions of thin layers of 5Y-PSZ suspensions are exposed to UV illumination, their vicinities can also be photopolymerized due to UV scattering by ceramic particles, resulting in notable increase in dimensions. This broadening effect can be greatly influenced by many factors, including refractive index of ceramic particles and solid loading [38,39]. Thus, the degree of discrepancy between designed and photocured dimensions should be carefully characterized and controlled for individual ceramic suspensions to achieve high accuracies for dental crown applications. To this end, disk-shaped regions with different diameters (100–1000 μm) were photocured for 3 sec to completely photopolymerize 50-μm-thick layers of 5Y-PSZ suspensions. Figure 9B shows measured diameters of disks in accordance with their initial values and percent (%) discrepancies between measured and initial values. When designed diameters were smaller than 400 μm, relatively large discrepancies were observed presumably due to resolution of a DLP engine. However, percent discrepancy decreased considerably with an increase in diameter and then changed slightly. Thus, based on these measurements, initial dimensions of dental crowns could be determined reasonably.

### 3.6. Microstructures of as-Printed 5Y-PSZ

**Figure 10:**
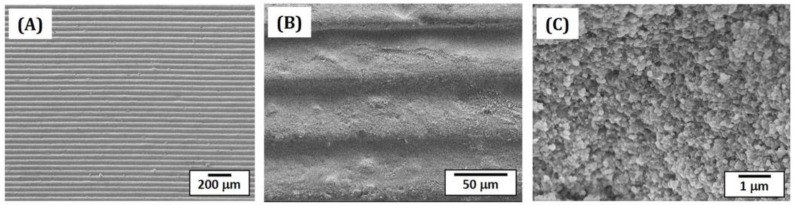
FE-SEM images showing free surfaces (**A**,**B**) and fracture surface (**C**) of as-printed 5Y-PSZ disks in the building direction.

Microstructures of as-printed 5Y-PSZ specimens were examined by FE-SEM, as shown in Figure 10A–C. Owing to the use of 50-μm-thick layers of a 5Y-PSZ suspension with UV illumination time of 3 s, specimens could be manufactured without notable defects (Figure 10A). In addition, photocured layers could be strongly bonded together without interfacial delamination (Figure 10B). The fractured surface showed that 5Y-PSZ particles could be distributed in photocured HDDA without notable aggregates or voids (Figure 10C).

### 3.7. Shrinkages and Relative Density of Sintered 5Y-PSZ

Following a tightly controlled debinding process with slow heating rates below 470 °C to remove photopolymerized HDDA and organic phases, 5Y-PSZ specimen and dental crowns were densified by sintering at 1500 °C for 2 h. Table 3 summarizes linear sintering shrinkages and relative densities of 5Y-PSZ disks. Linear shrinkages in diameter and thickness were 19.12 ± 0.18% and 19.65 ± 0.27%, respectively. It should be noted that these values should be carefully considered to design initial dimensions of dental crowns for the DLP process, which could offer high dimensional accuracy after sintering. Sintered 5Y-PSZ disks showed high densities (5.976 ± 0.023 g/cm^3^) measured by Archimedes’ principle, which corresponded to 99.03 ± 0.39% of theoretical density of 5Y-PSZ (6.034 g/cm^3^) [41]. However, it should be noted that theoretical density of 5Y-PSZ should be affected not by the contents of tetragonal and cubic phases, but also Y_2_O_3_ contents in both phases.

### 3.8. Microstructures and Grain Sizes of Sintered 5Y-PSZ

**Figure 11:**
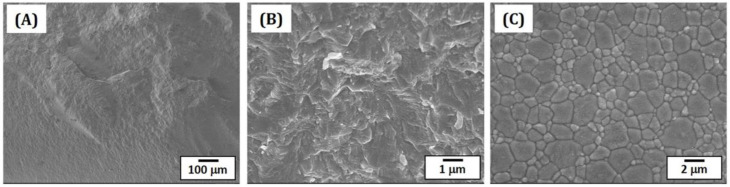
FE-SEM images showing fracture surfaces (**A**,**B**) and free surface of 5Y-PSZ disks after thermal etching at 1350 °C for 20 min (**C**).

Densification behavior and microstructures of sintered 5Y-PSZ specimens were characterized by FE-SEM, as shown in Figure 11A–C. Fractured surfaces of 5Y-PSZ specimens revealed high densification without notable defects, such as large voids and cracks (Figure 11A). In addition, no interfaces between layers were visible, suggesting strong interfacial bonding without imperfections, which is one of the most striking advantages of our DLP process using a specially designed recoating system. Only a few residual pores were observed (Figure 11B). To examine morphologies and of grains, the surfaces of specimens were polished and then heat-treated at 1350 °C for 20 min to reveal grain boundaries via thermal etching. Figure 11C show a representative FE-SEM image. Two different types of grains (large and small grains) were observed due to co-existence of tetragonal and cubic phases.

**Figure 12:**
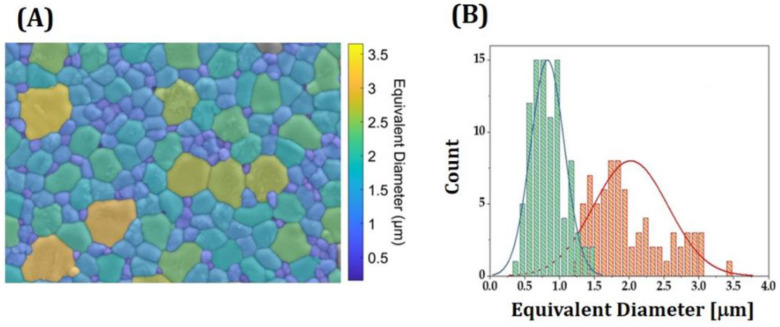
(**A**) Representative colored image reconstructed from a FE-SEM image showing grains with different colors and (**B**) counts as a function of equivalent diameter. The pink and green colors in Figure 12B represent size distributions of large and small grains, respectively.

Grain sizes were measured via the linear intercept method [54]. Values measured by considering all grains were 1.45 ± 0.18 μm. To roughly examine a distribution of grain sizes, an image analysis program was used. Figure 12A show a representative colored image reconstructed from a FE-SEM image, where different colors represent different sizes. Counts as a function of equivalent circle diameter—diameter of a circle closely matching the perimeter of a grain [55]—are shown in Figure 12B. Roughly calculated sizes of large (pink color) and small grains (green color) representing were 1.58 μm and 0.73 μm, respectively. It should be noted that large and small grains correspond to cubic and tetragonal phases, respectively.

It should be noted that densification behavior and crystalline phases (e.g., tetragonal and cubic phases) of 5Y-PSZ are strongly affected by sintering conditions such as temperature and dwelling time, thus resulting in different mechanical and optical properties. In addition, sintering conditions should be optimized individually for different particles since they have different particle sizes and specific surface areas. For example, it was reported that the highest hardness and fracture toughness were obtained after sintering at 1450 °C for 2 h [56]. Meanwhile, other reports demonstrated that a higher sintering temperature of 1550 °C was used to achieve high mechanical properties [57] and high optical translucency [58]. Thus, a further study is required to examine effect of sintering conditions (temperature and dwell time) on densification, grain growth, and crystalline phases of 5Y-PSZ manufactured using our DLP technique, in order to optimize mechanical properties and optical transmittance simultaneously for dental crown applications.

### 3.9. Crystalline Phases of Sintered 5Y-PSZ

**Figure 13:**
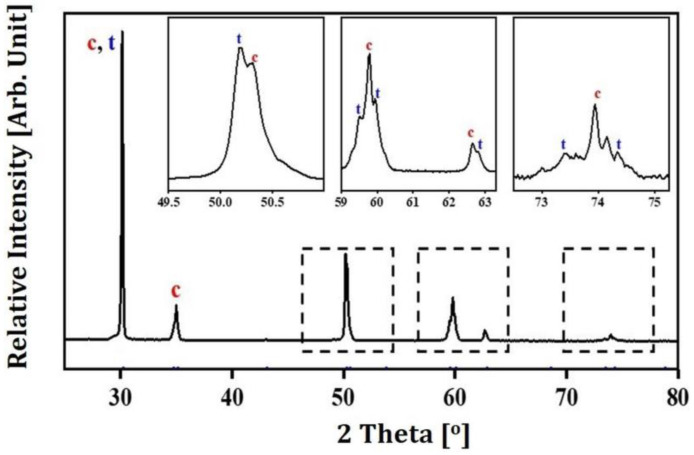
Representative XRD pattern of a 5Y-PSZ specimen sintered at 1500 °C for 2 h. “t” and “c” indicate tetragonal and cubic phases, respectively.

Crystalline phases of sintered 5Y-PSZ specimens were characterized by XRD. As expected, two different types of peaks corresponding to tetragonal (JCPDS card no. 01-079-1767) and cubic (JCPDS card no. 01-081-1550) phases were observed, as shown in Figure 13. Contents of tetragonal and cubic phases computed by Rietveld refinement using X’Pert Highscore Plus software were ~40.9% and ~59.1%, respectively. Measured values were comparable to those of commercial 5Y-PSZ [37]. This finding suggests that 5Y-PSZ dental crowns manufactured using our DLP process could offer reasonable translucency when they are highly densified without notable defects. It should be noted that cubic phase content can be further controlled by adjusting sintering temperature if necessary [43,59].

### 3.10. Chemical Compositions of Tetragonal and Cubic Grains

Chemical compositions of two different types of grains with tetragonal and cubic phases were characterized by EDS spot analyses. Table 4 summarizes the contents of elements (Zr, Y, and O) taken of large and small grains. Large grains showed higher Y_2_O_3_ contents (7.0 ± 0.3%) than small grains (3.8 ± 0.3%). This finding suggests that large and small grains have cubic and tetragonal phases, respectively.

### 3.11. Mechanical Properties of Sintered 5Y-PSZ

**Figure 14:**
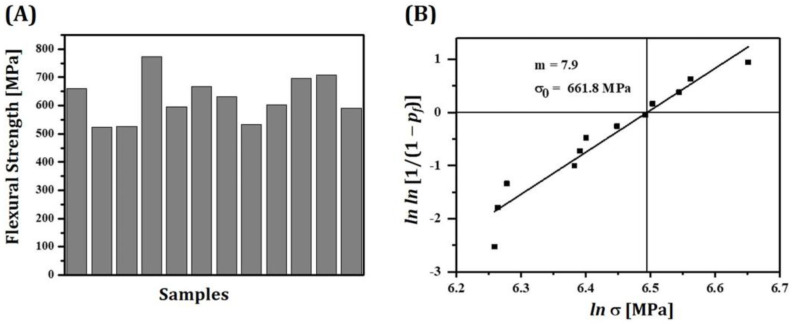
(**A**) Measured flexural strengths of 5Y-PSZ specimens and (**B**) Weibull plot of flexural strength.

For clinical applications, 5Y-PSZ dental crowns manufactured using our DLP process should have reasonable flexural strengths to withstand applied loads during their functions [40]. Thus, flexural strengths of 5Y-PSZ specimens were measured by three-point flexural strength tests. Twelve specimens were tested to calculate mean with standard deviation. All specimens showed reasonably high flexural strengths (625.4 ± 75.5 MPa), as shown in Figure 14A. These values were comparable to those obtained using dental CAD/CAM [37,59]. In addition, all specimens showed flexural strengths higher than 500 MPa. This finding suggests that our DLP technique using highly concentrated 5Y-PSZ suspensions with a solid loading of 50 vol% can manufacture not only single-unit prostheses (dental crowns) but also three-unit prostheses (dental bridges) [42].

Mechanical reliabilities of 5Y-PSZ specimens were evaluated by computing Weibull modulus (*m*) and characteristic strength (*σ_o_*) from a Weibull plot [45]. Figure 14B shows a Weibull plot of measured flexural strengths. *m* is a measure of the distribution of flexural strengths. Thus, for dental crown applications, 5Y-PSZ should have reasonably high *m* to provide high mechanical reliability with small deviations of flexural strengths and long-term mechanical function without failure. Computed *m* value of 5Y-PSZ was 7.9, which was comparable to those obtained by SLA process [22] and dental CAD/CAM [37]. On the other hand, *σ_o_* can be defined as a level of strength at which the *P_f_* becomes 63.2% and represents a statistical indicator of overall strength of the material. Thus, higher *σ*_0_ values are required for dental crown applications. Computed *σ_o_* of 5Y-PSZ was 661.8 MPa, which was comparable to those obtained by dental CAD/CAM [37]. From these *m* and *σ_o_* values, failure probability (*P_f_*) of 5Y-PSZ dental prostheses under flexural stress (*σ*) could be computed with the following equation

*P_f_* = 1 − exp[−(*σ*/*σ_o_*)^*m*^] (1)

When a bending stresses of 300 MPa was applied, *P_f_* value was as low as 0.0019. This finding suggests that single crowns made of 5Y-PSZ using our DLP techniques would not break during their functions under normal conditions.

### 3.12. Optical Translucency of 5Y-PSZ Disks

**Figure 15:**
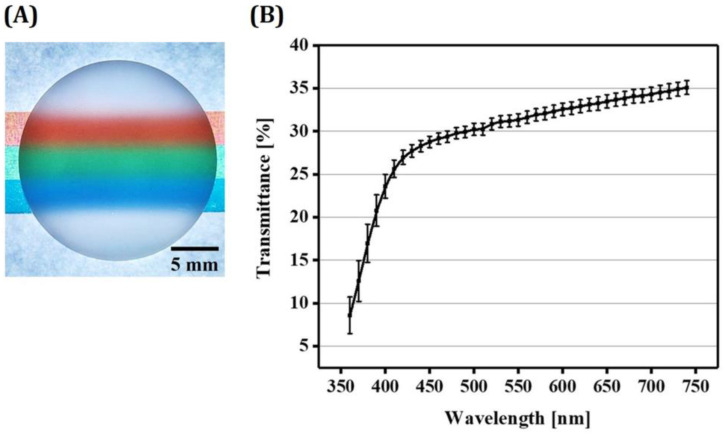
(**A**) Representative optical image of a 5Y-PSZ disk placed on a paper with colored lines and (**B**) spectral transmittance curve for sintered 5Y-PSZ disks.

For clinical uses, dental crowns should have reasonable optical translucency [35,59,60,61]. Thus, light transmittance capabilities of 5Y-PSZ specimens manufactured using our DLP technique was roughly evaluated. For these evaluations, 5Y-PSZ disks with a thickness of 1 mm were placed onto papers with colored marks, as shown in Figure 15A. The 5Y-PSZ disk clearly revealed colored marks, suggesting its reasonably high light transmittance capability attributed to high densification with negligible defects [6]. Optical transmittances of 5Y-PSZ disks were quantified by spectrophotometry. Results are displayed in Figure 15B. Three disks were tested to obtain mean and standard deviation. Spectral transmittance data were collected at 10 nm intervals from 360 nm to 740 nm. Percentage transmittance values computed to according to the standard (CIE standard illuminant D65) [46] were 31.4 ± 0.7%, which would be proper for some clinical uses. However, these values were lower than those obtained using dental CAD/CAM due to lower densification. Thus, enhancing densification behavior of zirconia, for example, by increasing solid loading in zirconia suspensions and/or using new sintering processes (e.g., microwave sintering [62] and two-step sintering [63]), still remains as one of the most challenging tasks in DLP of zirconia for dental applications [6].

### 3.13. Dimensional Accuracy of 5Y-PSZ Dental Crowns

**Figure 16:**
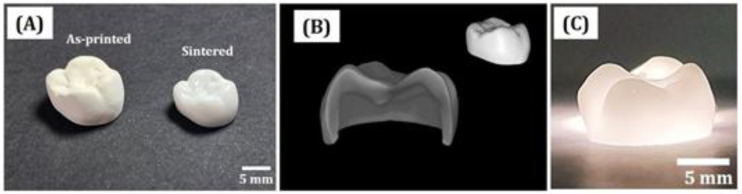
(**A**) Optical image showing 3D geometries of as-printed and sintered dental crowns, (**B**) representative μ-CT image of a sintered dental crown, and (**C**) optical image showing translucency of a sintered dental crown under light illumination.

To demonstrate utility of our DLP technique for dental applications, single crowns were manufactured using 5Y-PSZ suspensions. Their dimensional accuracies were then evaluated in terms of marginal and internal discrepancies [13,47]. A CAD model of a single crown was employed as a proof of concept (c.f., Figure 2B). To achieve high dimensional accuracy, initial dimensions of dental crowns were carefully determined by considering both enlargement by photopolymerization and shrinkage by sintering. In other words, initial dimensions were oversized than target dental crowns by a factor of ~25.2% and ~24.5% in the x-y and z directions, respectively. Figure 16A shows representative OM images of as-printed and sintered 5Y-PSZ dental crowns. Remarkable decrease in dimensions was observed due to high densification of 5Y-PSZ particles during sintering at 1500 °C for 2 h. However, μ-CT analyses revealed that no noticeable defects, such as voids, cracks or severe distortion, were observed for dental crowns even with sophisticated geometries (Figure 16B). In addition, dental crowns were translucent under light illumination (Figure 16C). It is reasonable to suppose that these dental crowns can have optical transmittances close to those of 5Y-PSZ disks (31.4 ± 0.7%), suggesting their potential for clinical uses.

**Figure 17:**
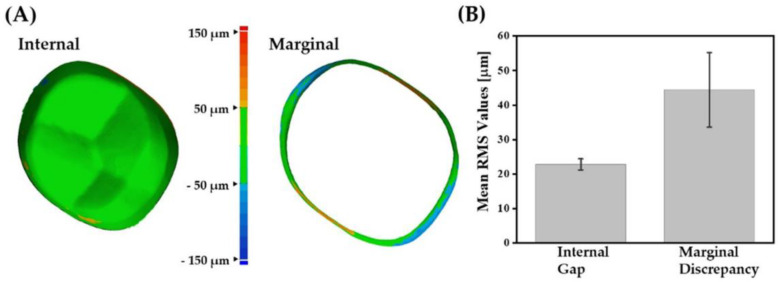
(**A**) Representative color maps generated by superimposition of scanned data of a 5Y-PSZ dental crown and original CAD data and (**B**) measured mean RMS values for marginal discrepancy and internal gap.

For offering long-term clinical success, dental crowns should have high dimensional accuracy (e.g., marginal gap ≤ 120 μm and internal gap ≤ 300 μm) [64]. Thus, 3D scanning technique was employed to perform quantitative analyses with high reliability and reproducibility [48,50]. For these evaluations, color maps were generated by superimposing scanned data of 5Y-PSZ dental crowns over an original CAD data for a target crown. Representative color maps are shown in Figure 17A. Most regions of internal surfaces showed green color, indicating good internal fit with a small discrepancy within ±50 μm. On the other hand, orange and blue colors were also observed at the marginal position. Mean RMS values for the internal gap and marginal discrepancy were 22.8 ± 1.6 μm and 44.4 ± 10.8 μm, respectively (Figure 17B). These values are comparable to those obtained for zirconia dental crowns manufactured using DLP [11,12]. These findings suggest that our DLP technique can offer reasonably high dimensional accuracy with assistance of tightly controlled processing parameters, including initial dimensions of dental crowns, sequential photopolymerization process, and heat-treatment (debinding and sintering).

## 4. Conclusions

5Y-PSZ dental crowns with desired mechanical properties, optical translucency and dimensional accuracy for clinical uses could be manufactured by carefully controlling processing parameters for our DLP process. The use of a high solid loading of 50 vol% in 5Y-PSZ suspensions allowed sintered 5Y-PSZ to have high relative densities (99.03 ± 0.39%), thus offering high flexural strength (625.4 ± 75.5 MPa) and % transmittance (31.4 ± 0.7). In addition, high dimensional accuracy (RMS for marginal discrepancy = 44.4 ± 10.8 μm and RMS for internal gap = 22.8 ± 1.6 μm) was achieved by carefully designing initial dimensions of dental crowns and photocuring time for 3D printing.

## Figures and Tables

**Table 1:** Constituents of 5Y-PSZ suspensions with a solid loading of 50 vol% used for our DLP process.

Role	Material	Weight [g]	Volume Percent (%)
Zirconia Particle	5 mol% yttria-partially stabilized zirconia (Zpex Smile)	70.75	50.00
Photocurable Monomer	1,6-hexanediol diacrylate (HDDA)	6.50	27.18
Diluent	Decalin	3.50	16.70
Dispersant	Solution of a structured acrylate copolymer with pigment-affinic groups (DISPERBYK-180)	1.42	5.60
Photo initiator	Diphenyl(2,4,6-trimethylbenzoyl) phosphine oxide (TPO)	0.13	0.49

**Table 2:** Schedule for multi-step debinding with slow heating rates and sintering at 1500 °C for 2 h.

Step	1	2	3	4	5
Heating Rate (°C/min)	2	0.8	0.8	1	5
Temperature (°C)	310	380	430	470	1500
Dwelling Time (min]	180	180	60	60	120

**Table 3:** Linear sintering shrinkages and relative densities of 5Y-PSZ disks after sintering at 1500 °C for 2 h.

Linear Sintering Shrinkage (%)		Measured Density (g/cm^3^)	Relative Density (%)
Diameter	Thickness		
19.12 ± 0.18	19.65 ± 0.27	5.976 ± 0.023	99.03 ± 0.39

**Table 4:** Chemical compositions of large and small grains corresponding to cubic and tetragonal phases characterized by EDS spot analyses.

	Elements (wt%)			Compositions (mol %)	
	Zr	Y	O	ZrO_2_	Y_2_O_3_
Large Grain	64.3 ± 0.9	9.5 ± 0.3	26.2 ± 1.0	93.0 ± 0.7	7.0 ± 0.3
Small Grain	66.8 ± 3.3	5.2 ± 0.8	28.0 ± 3.5	96.2 ± 0.7	3.8 ± 0.3

## Data Availability

Not applicable.
